# Operating for the Children

**Published:** 1883-08

**Authors:** E. C. Moore


### Operating for the Children.

BY DR. E. C. MOORE.

Before the Detroit Dental Society.

Mr. President and Gentlemen :--Having so recently retired from the honorable position of Secretary of this Society, and being impressed with the arduous duties of the office, I thought, to assist the present incumbent, (who, according to custom, should be the essayist for the evening), by presenting a few thoughts on the subject for our evening's discussion, as suggested by him at a previous meeting, that of the "management of children," who, at the time, may have dreamed, but little thought, of the lofty position to which he was so soon to be elevated, being then simply one of the rank and file of this august body; but, if he performs the duty of that office, he will be excused from the task of preparing a paper. This, like all other subjects mentioned for discussion before this or any other dental society, is a very important one, and, perhaps, like all others too, the *most* important one, but seriously, gentlemen, it is of great importance to know how to manage children. My experience in this direction is almost as varied as the number of children to whom I have been called to perform service; there are scarcely two dispositions alike, and each will require management in accordance with their peculiar characteristic traits or individuality, and the secret of success lies chiefly in the study of character and disposition, but we have often two instead of one to manage, and sometimes both parents to contend with, and their influence over the child, combined with the inherited cowardly disposition. This complicates matters sometimes to an exasperating degree, and generally ends in some plain talk of the "Dutch Uncle" order; then again, we are called upon to act, both in the capacity of parent and operator, while we are not allowed the full privilege of either. You will all understand the significance of this ; as a rule, I think I may say, that beyond the age of six years, we will accomplish our work with more satisfaction to ourselves and less discomfort to the little patient if left entirely alone. Parents will bring their child or children for us to do the very best thing possible with the view of development and preservation of the little one's teeth; they are extremely anxious in this regard, because, perhaps, it is an opportunity they did not enjoy during their juvenile years, and they are *sorely* impressed with the importance and necessity of good teeth, and are going to do their whole duty toward their children in this particular. By way of manifestation of their anxiety, they will allow the household duties to take care of themselves, or go entirely uncared-for, that they may remain with the little patient, when, I think, it would be safe to say, nine times out of ten the child would get along better if allowed to come alone, for at the first manifestation of pain, or even inconvenience, wearyness, or nervousness, they will begin to caress or sympathize with the child to such extent as to make it really think that they are most terribly and unnecessarily abused. This with some dispositions might work well enough, but I think with the majority it has quite the opposite result from that which we both desire. I have known them to go even so far as to drop upon their knees and pray, while the child was undergoing some minor operation, or under the effects of an anæsthetic, what for the good Lord only knows.

Then again, it does not do to carry firmness or sternness to the other extremity, and assume an expression as savage as a meat ax, but use all intermediate degrees of firmness or gentleness, as the peculiar disposition of the case in hand might indicate. Then it is apparent that the key note to success in the management of children lies in the art of reading and studying disposition, and the admixture of sympathy or gentleness with firmness. In this study we are often limited to a very short acquaintance, having, perhaps, never seen the child before coming to fulfill an appointment; but by practice we become very apt in these matters, and can learn a great deal of a child's disposition in a comparatively short space of time. To give you an instance: A child was left in my charge by her father as per engagement made by him some days previous, while he (the father) went about his own affairs (a very wise thing for him to do). After a few minutes chat with the child to get acquainted, I began to arrange appliances and materials handy to make the operation as short as possible, and while my back was turned for a few minutes in an adjoining room, on returning, to my astonishment, the room was empty of things animate. She had taken her hat and parasol and in silence fled, or, like the Arb, folded her tent and gently stole away; but, unlike the Arb, I did not stop for either tent or hat, but immediately went in pursuit, and about half a block distant overtook her, and a more astonished child imagination could scarcely picture. This was a case where a mild degree of firmness was required, I might, perhaps, better say, only simple earnestness; not that the child had a bad or wilful disposition—quite the contrary, as was subsequently proven—a sweet and unusually bright child, but the result of inefficient restraint or government at home. She had never known what home government was; in short, she was the supreme ruler in the household, and all simply because she could be. In this case it was only necessary to convince this child that what I said, I meant, and that that which I intended doing for her was for her own good, and ever after, I had not the slightest trouble, and she has not now the least fear or dread of me. You will see now, gentlemen, what a wise action on the part of the father not to have remained, for he could not, or would not, have controlled her in the least. I can imagine he would say, now, daughter, you do so and so, under threat, when he knew he had not the slightest intention of inflicting punishment, and she knew it full better than he.

The government of children, not only as dental practitioners, but the government of children in general in the family circle, is one of the important of all important studies, for, the future career and character so much depend upon the molding of the young mind—as the twig is bent so the tree inclines.

This is an important study to us as practitioners of our specialty, for like the molding of the young mind, so much of the future comfort and usefulness of the mouth and teeth depend upon their proper molding development and preservation, and all this to be accomplished at the proper time, and even more thoroughness if possible in operations is required than in the adult.

1. Because of the greater length of time, before them both for the enjoyment of their teeth, and the test of the thoroughness of the work.

2. Because the child passes, or is passing a period in life when, owing to the undeveloped condition of tooth structure, they are more likely to be attacked by decay or caries, and the condition much less able to resist such an attack than when fully matured; hence, I say, the importance of minuteness in the process of filling.

In the control of children while undergoing these disagreeable and often painful operations, an appeal to their nobler nature may be very effective, in fact is in a majority of cases, a most potent method; and I ask again your indulgence while I relate another recent experience demonstrating the potency of this method: A boy about eight years of age, and of noble birth, bearing unmistakable signs of elements of character susceptable of development into a man considerably above the mediocrity, both in intellect and nobleness of nature, was sent to me with a note from the mother, putting him in my charge, to do for him just as I would for one of my own family. He was given an appointment, which he fulfilled to the minute, notwithstanding the bleak winter day. During the earlier or preparatory stages of the process of filling, he evinced signs of fright and nervousness, not knowing just what was coming (having a few days previous undergone the severe operation of the removal of one of the first permanent molars), but having by this time so familiarized myself with his disposition, I felt sure that I could control him. I said to him, calling him by name: "How much older is your sister than you?"—knowing that she must be at least two or three years his junior. The query acted as if by magic. He at once, in an astonished look, said: "I am the older," and I, in return, in an apparantly surprised look at this information, replied, that I

thought she was the older, because she behaved so nicely in having her teeth filled. This was a settler, and had the desired effect, and henceforward I had no more trouble with him any more than if he had been an Egyptian mummy, although some of the operations must have been somewhat painful. It was plainly written in his countenance that his pride was touched.

There are no prscribed rules by which we can be governed in the management of children under these trying circumstances.

